# Synergistic Effects of Fe_2_O_3_ Nanotube/Polyaniline Composites for an Electrochemical Supercapacitor with Enhanced Capacitance

**DOI:** 10.3390/nano11061557

**Published:** 2021-06-13

**Authors:** Farkhod Azimov, Jihee Kim, Seong Min Choi, Hyun Min Jung

**Affiliations:** 1 Department of Advanced Materials Sciences and Engineering, Kumoh National Institute of Technology, 61 Daehak-ro, Gumi 39177, Korea; afara13@gmail.com; 2 Department of Applied Chemistry, Kumoh National Institute of Technology, 61 Daehak-ro, Gumi 39177, Korea; hhhhhh3021@gmail.com (J.K.); tjdals2351@naver.com (S.M.C.); 3 Department of Energy Engineering Convergence, Kumoh National Institute of Technology, 61 Daehak-ro, Gumi 39177, Korea

**Keywords:** α-Fe_2_O_3_ nanotube, supercapacitor, polyaniline, capacitance

## Abstract

α-Fe_2_O_3_, which is an attractive material for supercapacitor electrodes, has been studied to address the issue of low capacitance through structural development and complexation to maximize the use of surface pseudocapacitance. In this study, the limited performance of α-Fe_2_O_3_ was greatly improved by optimizing the nanotube structure of α-Fe_2_O_3_ and its combination with polyaniline (PANI). α-Fe_2_O_3_ nanotubes (α-NT) were fabricated in a form in which the thickness and inner diameter of the tube were controlled by Fe(CO)_5_ vapor deposition using anodized aluminum oxide as a template. PANI was combined with the prepared α-NT in two forms: PANI@α-NT-a enclosed inside and outside with PANI and PANI@α-NT-b containing PANI only on the inside. In contrast to α-NT, which showed a very low specific capacitance, these two composites showed significantly improved capacitances of 185 Fg^−1^ for PANI@α-NT-a and 62 Fg^−1^ for PANI@α-NT-b. In the electrochemical impedance spectroscopy analysis, it was observed that the resistance of charge transfer was minimized in PANI@α-NT-a, and the pseudocapacitance on the entire surface of the α-Fe_2_O_3_ nanotubes was utilized with high efficiency through binding and conductivity improvements by PANI. PANI@α-NT-a exhibited a capacitance retention of 36% even when the current density was increased 10-fold, and showed excellent stability of 90.1% over 3000 charge–discharge cycles. This approach of incorporating conducting polymers through well-controlled nanostructures suggests a solution to overcome the limitations of α-Fe_2_O_3_ electrode materials and improve performance.

## 1. Introduction

As electrical energy storage devices, electrochemical supercapacitors have the characteristics of high power, fast charge–discharge, high cycle efficiency, and stability. These characteristics cannot be fully realized in current lithium-ion battery technology; therefore, the importance of the energy storage role of the supercapacitor in various mobile devices is increasing. However, the limit of the energy storage density is always an obstacle to allow the supercapacitor to be the main storage device. This is due to the basic principle that supercapacitors directly store electric charge on the electrode surface. This principle results in high power, but also causes low energy storage density [1,2]. Therefore, the development of an electrode material with high charge storage capacity and the creation of an electrode with maximum surface area through the formation of a nanostructure are becoming the main development goals for supercapacitors. Hence, MnO_2_ [3,4,5,6], NiO [7,8], and RuO_2_ [9,10]-based materials have been developed to exhibit pseudocapacitance with high charge storage capacity; thus, beginning to overcome the limitations of low-capacity carbon-based electrode materials that depend on electrical double-layer capacitance [11,12]. Among the electrode materials of such pseudocapacitive transition metal oxides, iron oxides are promising candidates that are inexpensive, eco-friendly, and have abundant raw material resources suitable for mass use; however, they have not received much attention because they present a relatively small specific capacitance and inherent low electrical conductivity [13].

Recently, studies on the application of iron oxide electrode materials have been reported, suggesting methods to overcome the iron oxide limitations for electrodes. In particular, α-Fe_2_O_3_ has been extensively used in many fields such as catalysts, pigments, and sensors [14]. Various nanostructure manufacturing technologies have been developed and these technologies have been applied to electrode material manufacturing, resulting in improved performance. Nanostructures such as nanowires [15], nanorods [16], nanoporous structures [17], nanoflowers [18], and nanotubes [19,20,21] have been fabricated and applied to supercapacitors; the fabrication methods include direct electrodeposition on the electrode [22] and hydrothermal [23] and templating methods [24]. The application of α-Fe_2_O_3_ in various nanostructures maximizes the surface where the electrolyte and reversible redox faradaic reaction occurs and increases contact with the current collector, enabling effective use of the generated electric charge [25]. Therefore, it is believed that the nanotube shape can provide an optimal structure among other nanostructures. If the shell thickness of the tube can be greatly reduced by maximizing the surface area while maintaining the nanostructure, the charge development can be maximized on the inner and outer surfaces of the tube [26]. However, when the electrical conductivity of the material itself is low, a structure in which one axial direction is lengthened, such as nanotubes and nanorods, may have an opposite effect on the effective use of the generated charge by transferring it to the current collector. Therefore, in the case of α-Fe_2_O_3_ and MnO_2_ nanomaterials with low electrical conductivity, combinations of highly conductive materials have generally been applied, and the use of carbon nanotubes, graphene, and conducting polymers has been reported [27].

Among the recent examples of Fe_2_O_3_ nanotube applications as supercapacitor electrodes, the use of ZnO nanowire arrays as sacrificial templates for Fe_2_O_3_ nanotube synthesis has been reported [28]. In this case, the asymmetric supercapacitor was composed of Fe_2_O_3_ nanotubes and MnO_2_ nanowires, and a specific capacitance of 197 Fg^−1^ was obtained. In addition, the fabrication of α-Fe_2_O_3_ nanotube arrays by the electrochemical anodization of Fe-foil and their specific capacitance of 138 Fg^−1^ as an electrode material was reported [20]. From these results, it can be said that the application of a nanotube structure of Fe_2_O_3_ is quite effective for improving capacitance. As an approach to overcome the limitations of low-conductive α-Fe_2_O_3_ nanotubes, a study on enhanced performance through combinations with reduced graphene oxide (rGO) has been reported [19]. In this case, a two-dimensional conductive pathway was formed by anchoring the α-Fe_2_O_3_ nanotubes to the rGO. Specific capacitance of 181 Fg^−1^ was obtained, and it was reported that the increasing specific capacitance was sevenfold higher than that of the case using α-Fe_2_O_3_ nanotubes alone. Nevertheless, in the results reported thus far, there has not been a study on the combination of Fe_2_O_3_ nanotubes and conducting polymers that improves the performance by overcoming the low conductivity caused by the nanotube structure and intrinsic low conducting Fe_2_O_3_. In this study, the aim was to combine the nanoscale coating of polyaniline (PANI) with α-Fe_2_O_3_ nanotubes to improve the performance for a supercapacitor electrode. In addition, a novel method for the fabrication of α-Fe_2_O_3_ nanotubes was proposed, which provided the optimized nanotube structures for combination with PANI. In the ZnO template method for Fe_2_O_3_ nanotubes, in order to control the inner diameter of the tube and the thickness of the tube shell, the ZnO nanowire array template must be properly adjusted and prepared; this required finding a new manufacturing condition [28]. Electrochemical anodization is a very effective method for constructing a nanotube array [29], although it is not suitable for the formation of individual α-Fe_2_O_3_ nanotubes for PANI coatings. In particular, to realize the performance through combination with PANI, it is important to control the inner diameter and wall thickness of the tube so that the PANI coating can be effectively applied inside and outside the tube.

Here, as a method of manufacturing α-Fe_2_O_3_ nanotubes, evaporated Fe(CO)_5_ was used as an iron oxide source, and porous anodized aluminum oxide (AAO) was used as the template. Porous AAO can be a very convenient manufacturing base because its pore size and shape control technology is standardized, and AAO is mass-manufactured and commercially available. However, the issue arises as to whether uniform iron deposition is possible on the AAO template. In this study, it was discovered that evaporated Fe(CO)_5_ decomposed very selectively on the alumina surface, which became the basis for iron nanotube fabrication. Based on this, PANI can be coated inside and outside the nanotubes, as a structure that optimally utilizes the surface pseudocapacitance of electrode materials. Through this α-Fe_2_O_3_ nanotube/PANI complexation, a synergistic effect was achieved that dramatically increased the capacitance, which was significantly low in the pure nanotube form.

## 2. Materials and Methods

### 2.1. Materials

Iron (0) pentacarbonyl (>99%), ammonium persulfate (>98.0%), aq. HCl (>37%), sodium hydroxide (>98%), *N*-methyl-2-pyrrolidone (NMP) (anhydrous 99.5%), and aniline (>99%) were purchased from Sigma-Aldrich (Yongin, Korea). Anodic aluminum oxide (AAO) (Whatman^®^ Anodisc 47, 47 mm diameter, 60 µm thickness, and 200 nm pore size) was purchased from Merck (Seoul, Korea).

### 2.2. Synthesis of α-Fe_2_O_3_ Nanotubes

The α-Fe_2_O_3_ nanotubes were fabricated via Fe(CO)_5_ vapor deposition using AAO as the template. Nanotubes were grown on AAO by the deposition of Fe(CO)_5_ at different exposure times of 1–12 h in a glass vessel, to which a 47 mm diameter AAO disc was attached at the top, 4 mL of Fe(CO)_5_ was placed on the bottom, and the temperature was maintained at 25 °C. After 1–12 h of deposition, the Fe-deposited AAO was treated by air-oxidation at 400 °C for 2 h. The AAO template was removed by immersing it in 1 M aq. NaOH solution. The resulting solid was isolated by centrifugation and continuously washed with deionized water and ethanol, and then dried in a vacuum oven at 110 °C for 8 h.

### 2.3. Polyaniline (PANI) Particles Synthesis

PANI was synthesized according to a previously reported method [30]. Aniline, (1.86 g, 0.02 mol) was dissolved in 50 mL of 1 M HCl in a 250 mL round-bottomed flask and sonicated. Next, 4.56 g (0.02 mol) of ammonium persulfate (APS) was added to the reaction mixture and refrigerated at 0 °C for 2 h. APS/HCl solution with aniline mixture was polymerized at 0 °C. The synthesized PANI was washed with deionized water and ethanol, and dried under vacuum.

### 2.4. Synthesis of PANI@α-NT-a

First, 186 mg (2.0 mmol) of aniline in 5 mL of 1 M HCl aq. solution was added to 50 mg of α-NT. Then, 456 mg (2.0 mmol) of APS in 5 mL of 1 M HCl aq. Solution, which was refrigerated to 0 °C, was added into α-NT/aniline mixture solution. After reaction for 2 h at 0 °C, the polymerized PANI on the α-NT mixture was separated by centrifugation. The resulting product was washed with deionized water and ethanol, and dried under vacuum. The final product, obtained as α-NT combined with PANI, was named PANI@α-NT-a.

### 2.5. Synthesis of PANI@α-NT-b

The α-Fe_2_O_3_/AAO disc (α-NT before removal of the AAO template) was immersed into 626 mg (6.72 mmol) of aniline in 5 mL of 1 M HCl aq. solution. Afterwards, 1.53 g (6.72 mmol) of APS in 5 mL of 1 M HCl, which was refrigerated to 0 °C, was added to the prepared mixture solution. After a 2 h reaction time at 0 °C, the polymerized PANI in the α-Fe_2_O_3_/AAO disc was removed from the reaction mixture. The AAO template was removed from the resulting α-Fe_2_O_3_/AAO disc by immersing it in 2 M aq. phosphoric acid solution for 1 h. The product was separated by centrifugation. The resulting product was washed with 0.1 M HCl aq. solution, deionized water and ethanol, and dried under vacuum. The product, obtained as α-NT combined with PANI, was named PANI@α-NT-b.

### 2.6. Morphological and Structural Characterization

The microscopy studies were carried out using field emission scanning electron microscopy (FE-SEM) (Tescan MAIA 3 LM) and transmission electron microscopy (TEM) (Jeol JEM-2100), and the crystal structures were analyzed using an X-ray diffractometer (XRD) (SmartLab) with Cu–Kα radiation (λ = 1.5406 Å). Thermogravimetric analysis was performed using an Auto-TGA Q500 instrument (TA Instruments). Raman analysis was performed using System 1000 (Renishaw). The Brunauer–Emmett–Teller (BET) specific surface area and pore size distribution were measured by nitrogen adsorption and desorption experiments at −196 °C using an accelerated surface area and porosity analyzer (ASAP 2420, Micromeritics).

### 2.7. Electrochemical Measurements

The working electrodes were prepared by mixing 70% active materials, 20% carbon black, and 10% polyvinylidene fluoride. Then, NMP was added dropwise and stirred to prepare slurry. The resulting slurry was coated onto the Ni foam, which was followed by drying at 60 °C for 12 h in a vacuum oven.

The electrochemical properties of electrode materials were investigated with cyclic voltammetry (CV), galvanostatic charge/discharge (GCD) and electrochemical impedance spectroscopy (EIS) analyses, using AUTOLAB PGSTAT 30 apparatus. The electrochemical performance of all electrodes was evaluated using an electrochemical workstation composed of a typical three-electrode system, and electrochemical tests were performed in a 1 M Na_2_SO_4_ aqueous electrolyte. All electrochemical tests, including CV and GCD tests, were carried out from 0.0 to 1.0 V. EIS was performed in a frequency range of 10^−2^ to 10^5^ Hz with an amplitude of 5 mV. The cycling activity was evaluated by continuous cyclic voltammetry at a scan rate of 10 mV^−1^ for over 1000 cycles. The gravimetric specific capacitance was calculated from the GCD and CV curves according to Equations (1) and (2):

(1) $$C_{p}=\frac{Q}{\Delta V\times m}=\frac{I\times t}{\Delta V\times m}$$

(2) $$\;C_{p}=\frac{A}{2mk\left(V2-V1\right)}$$

where *I* is the current applied (*A*), *t* is the discharge time (s), Δ*V* is the discharge potential range (V), *m* is the mass of the electrode material used, *A* represents the discharge current, and (*V2* − *V1*) is the discharge voltage variation.

## 3. Results and Discussion

### 3.1. Fabrication of α-Fe_2_O_3_ Nanotube and Polyaniline-Combined Structures

**Scheme 1:**
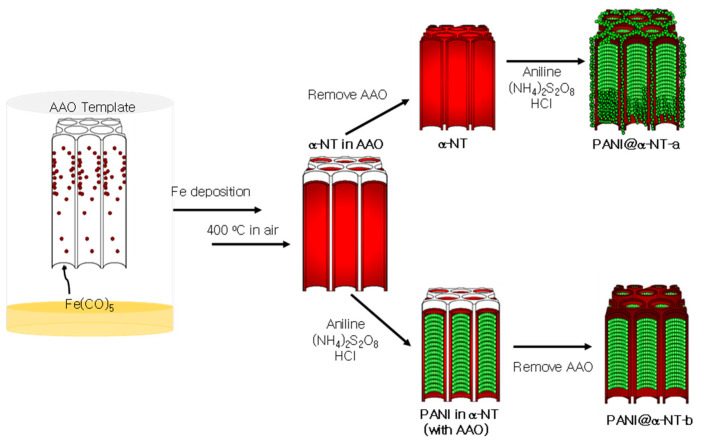
Schematic illustration of α-Fe_2_O_3_ nanotube (α-NT), PANI@α-NT-a, and PANI@α-NT-b fabrication.

**Figure 1:**
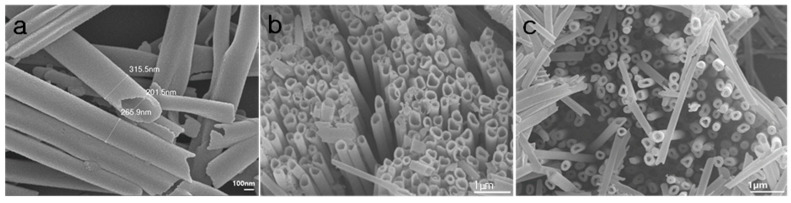
SEM images of (**a**,**b**) α-Fe_2_O_3_ nanotube (α-NT) from 3 h deposition of Fe(CO)_5_, and (**c**) α-NT from 12 h deposition of Fe(CO)_5_.

The α-Fe_2_O_3_ nanotubes were devised to maximize pseudocapacitance and utilize the surface electrical charge by combining them with polyaniline (PANI) (Scheme 1). It was found that Fe(CO)_5_ selectively decomposed on the alumina surface to form metallic iron, and this enabled the fabrication of α-Fe_2_O_3_ nanotubes with a precisely controlled tube thickness and diameter. First, the Fe nanotubes were fabricated using porous AAO as a template and Fe(CO)_5_ vapor deposition as the metallic iron layer in AAO pores with a length of several micrometers and a wall thickness of 20–30 nm. Subsequently, in the process of air oxidation and removal of AAO, individually separated α-Fe_2_O_3_ nanotubes with a wall thickness of 20–30 nm and a diameter of 150–170 nm were formed (Figure 1). This was used in the composites with PANI (Scheme 1). In the formation of nanotubes using Fe(CO)_5_ deposition, the wall thickness was controlled by the deposition time. The nanotube wall continuously increased for up to 3 h and grew to a thickness of 20–30 nm. After 3 h of deposition, the growth rate was inhibited, and the thickness reached 60 nm after 24 h. The sudden slowdown of deposition after reaching a certain thickness indicated that the Fe(CO)_5_ selectively decomposed on the alumina surface. This is the reason for obtaining a tube with a thin and uniform thickness, regardless of the tube length. Considering the electrochemical capacitor application, it was advantageous to form the thinnest tube wall that allowed mechanical stability; therefore, a 3 h deposition process with a stable tube structure formation was suitable. The BET surface area of α-Fe_2_O_3_ nanotubes obtained from 3 h deposition was 85 m^2^ g^−1^ and that from 12 h deposition was 33 m^2^ g^−1^; as the nanotubes increased in wall thickness, the surface area also decreased as expected.

**Figure 2:**
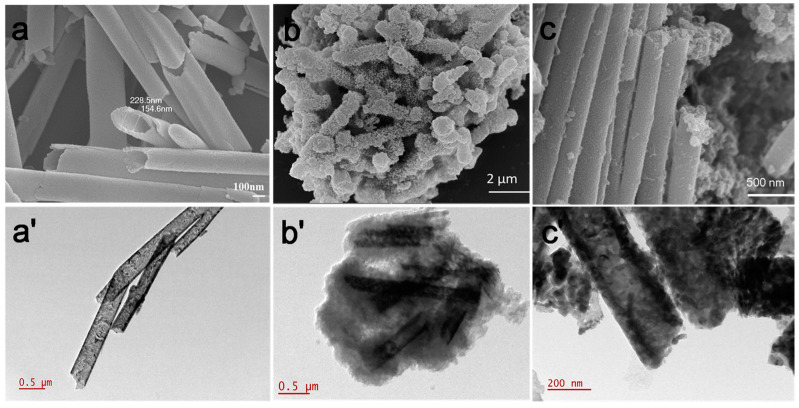
SEM and TEM images of the prepared PANI@α-NTs; (**a**,**a′**) α-NT, (**b**,**b′**) PANI@α-NT-a, (**c**,**c′**) PANI@α-NT-b.

The prepared α-Fe_2_O_3_ nanotubes (α-NT) were combined with PANI in two ways (Scheme 1). The first method was to polymerize PANI in an aniline reaction solution containing α-NT to form PANI on the inside and outside of the nanotube (PANI@α-NT-a). The second method was to selectively form PANI inside the nanotubes (PANI@α-NT-b). In this method, aniline enters the nanotube pore by capillary action and polymerizes to form PANI inside the nanotube before the AAO is removed. The prepared PANI@α-NT-a and PANI@α-NT-b were observed using SEM and TEM (Figure 2). From the SEM images of PANI@α-NT-a, PANI covered the entire surface while maintaining the rod shape of α-NT. It was observed that PANI was selectively coated on α-NT, which may be the result of the positive charge character of PANI being combined through interaction with the negatively charged iron oxide surface (Figure 2b). The TEM image showed that the α-NTs were surrounded by PANI and maintained their original tube structure (Figure 2b′). In the SEM and TEM images of PANI@α-NT-b, PANI coating on the outside of the nanotube was not observed, and the presence of PANI filling the inside was confirmed by comparing the images of the pristine α-NT (Figure 2c,c′).

**Figure 3:**
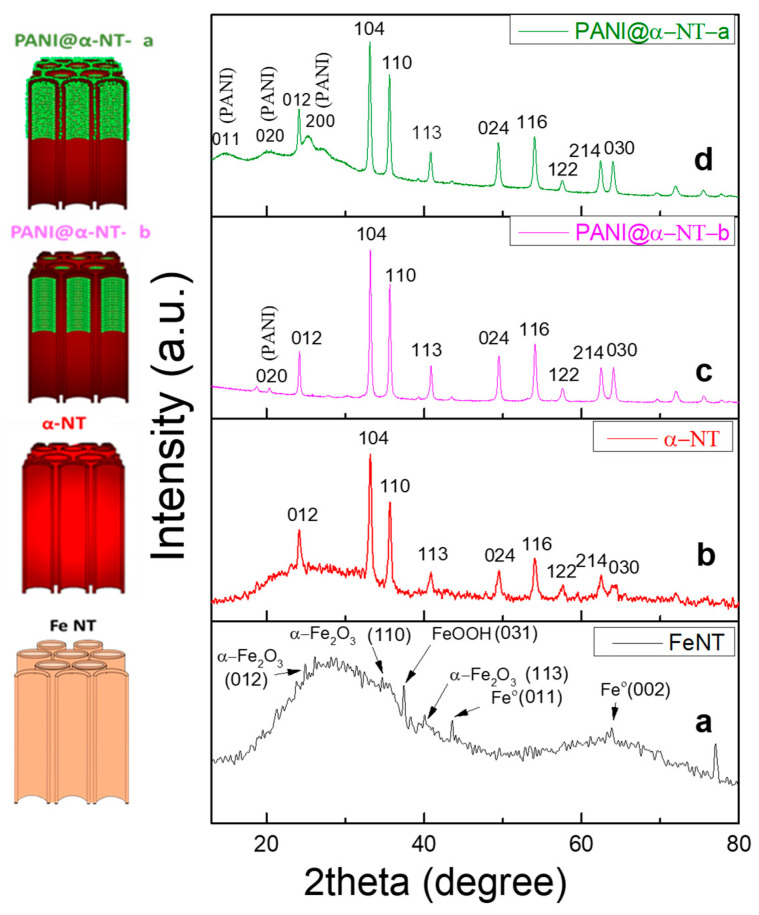
XRD analysis of (**a**) FeNT, (**b**) α-NT nanotubes, (**c**) PANI@α-NT-b, and (**d**) PANI@α-NT-a.

**Figure 4:**
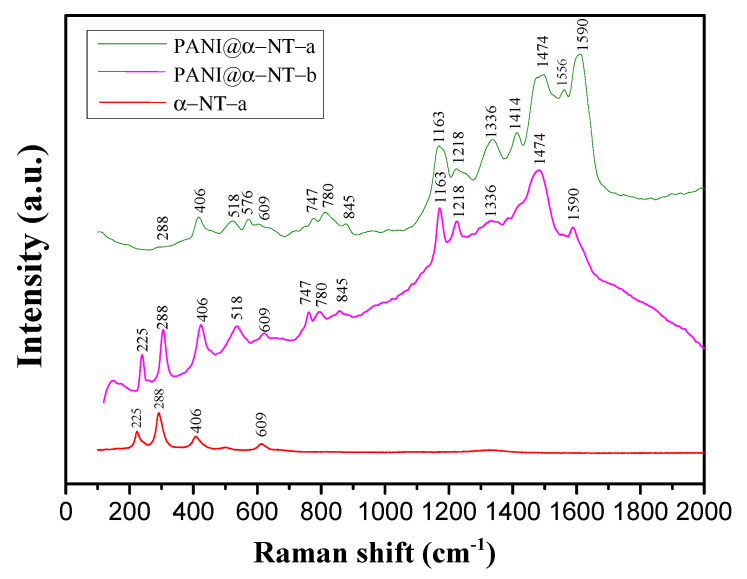
Raman spectra of PANI@α-NT-a, PANI@α-NT-b, and α-NT.

The phase and composition of the PANI@α-NT composites from each preparation step were characterized using XRD (Figure 3). The XRD pattern in Figure 3a shows the mixed iron sates of nanotube (FeNT); it seems that Fe_2_O_3_ and FeOOH were formed from partial oxidation during AAO removal under aq. NaOH conditions, including Fe(0) firstly formed by decarbonylation of Fe(CO)_5_. After thermal oxidation on FeNT, all the diffraction peaks for the formed α-NT were well indexed to the hexagonal α-Fe_2_O_3_ (JCPDS 33-0664) (Figure 3b). The nanotubes impregnated with PANI (PANI@α-NT-a) showed additional XRD peaks corresponding to PANI (JCPDS 06-0464), which were 14.5°, 20.4°, and 25.4° (011, 020, 200) (Figure 3d). These peaks all corresponded to the crystal plane of PANI. In the case of PANI@α-NT-b, very weak diffraction peaks for PANI were detected because of the relatively smaller amount of PANI impregnated into the nanotube pores (Figure 3c). The amount of PANI in the PANI@a-NT composites was estimated using TGA (Figure S1). The PANI@α-NT-b composites exhibited a lower weight loss of 10.1% relative to PANI@α-NT-a, where 77.8% of the weight was lost at 800 °C. This indicates that PANI@α-NT-a had a relatively higher PANI composition, which covered the α-NT surfaces at 100–200 nm thickness, and PANI@α-NT-b had a lower PANI ratio (10%), which was only incorporated in the pores of α-NT. The Raman spectra of PANI combined with α-NT are shown in Figure 4: the peaks observed at 1474–1590 cm^−1^ region correspond to the C–C and C=C stretching vibrations of benzenoid and quinoid rings. The peaks observed at 1336–1414 cm^−1^ are assigned to the C–N^+^ delocalized polaronic structure, which is characteristic of the protonated imine form of PANI in PANI@α-NT-b and PANI@α-NT-a. The band of C–N stretching vibrations can be observed at 1218 cm^−1^, and the C–H bending vibration of quinonoid rings in the PANI base is observed at 1163 cm^−1^. The bands at 845, 780, 747, and 518 cm^−1^ correspond the benzene-ring deformations of aromatic rings and out-of-plane benzene ring vibrations of the protonated emeraldine form of PANI. The peaks in the region 609, 406, 288 and 225 cm^−1^ correspond to Fe–O bands from α-NT [31,32]. Some of the Fe–O bands, such as 288 and 225 cm^−1^, were weakened in the case of PANI@α-NT-a, which was interpreted as the result of PANI completely wrapping over the surface of α-NT.

### 3.2. Electrochemical Properties

**Figure 5:**
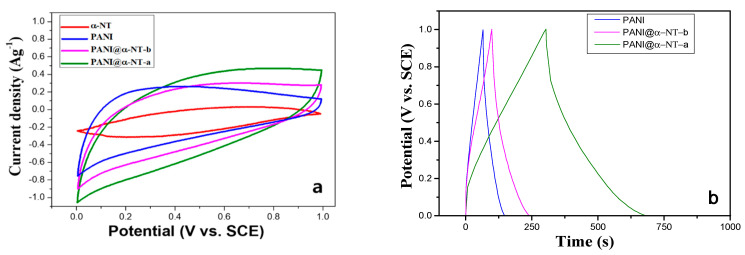
(**a**) Cyclic voltammograms of PANI@α-NTs and PANI. (**b**) Galvanostatic charge–discharge behavior of PANI@α-NTs and PANI at 0.5 Ag^−1^ current density.

Electrochemical investigations were performed on PANI@α-NTs. The α-Fe_2_O_3_ nanotubes, which are considered to exhibit pseudocapacitive behavior, showed low specific capacitance in the electrode composed alone without combining PANI. In the galvanostatic charge–discharge experiment, α-NT showed only 8.4 Fg^−1^ at a current density of 0.5 Ag^−1^. This is somewhat lower than the 10–20 Fg^−1^ which is generally observed in α-Fe_2_O_3_ nanoparticles [33], indicating the dependence on the morphology of the electrode materials due to the low electrical conductivity of α-Fe_2_O_3_. α-NT is a 1–3 μm long tube with a wall thickness of 20–30 nm and a diameter of 150–170 nm. If the electrode material conductivity itself is low, the use of electrical charge generated by the surface redox is limited by the charge transfer ability of the α-Fe_2_O_3_ material. As a result, it had a lower specific capacitance due to it having the shape of an elongated tube, which was disadvantageous for charge transfer and effective contact with the current collector, although α-NT had a larger BET surface area of 85 m^2^ g^−1^ than α-Fe_2_O_3_ nanoparticles with a surface area of 37 m^2^ g^−1^. Here, PANI@α-NTs were prepared in a form capable of overcoming the limitations of α-NT and exhibited synergistic effects in terms of conductivity by being combined with PANI. For PANI@α-NT-a and PANI@α-NT-b, their PANI contents were 78 wt.% and 10 wt.%, respectively, and the discharge capacitances measured at a current density of 0.5 Ag^−1^ were 185 Fg^−1^ and 62 Fg^−1^, respectively, showing a large difference between the two cases. More importantly, compared with the specific capacitance of 40 Fg^−1^ for pure PANI and 8.4 Fg^−1^ for α-NT, significantly increased capacitances were obtained for PANI@α-NTs (Figure 5 and Table 1). This is believed to be the result of a clear synergistic effect, not an arithmetic sum of the capacitances of the two components. Particularly, for PANI@α-NT-a, in which PANI surrounds the inside and outside, the effect of increasing the capacitance was very large, and the highest value was obtained. This 185 Fg^−1^ level of specific capacitance exceeds the highest levels of 100–140 Fg^−1^ obtained by α-Fe_2_O_3_ nanostructures directly growing on the electrode surface or composites with various carbon materials [34,35,36]. PANI@α-NT-b contained only 10 wt.% PANI, and PANI was selectively composited to exist inside the pores of α-NT. PANI was coated inside the pores, resulting in the imparting of conductivity over the low-conductive α-Fe_2_O_3_ material, increasing electrical charge formation on the α-Fe_2_O_3_ surface, or decreasing the loss of generated charge. These effects could be maximized with PANI@α-NT-a. PANI surrounded both the inside and outside of the nanotube, and all surfaces of α-Fe_2_O_3_ were in contact with the conductive PANI, minimizing electrically isolated parts that could not be utilized. Moreover, in the PANI@α-NT-a structure, the effect of conductive contact between each nanotube mediated by the coated PANI can also play an important role in capacity enhancement. In particular, the α-NT content in PANI@α-NT-a was 22 wt.%, and to represent the specific capacitance of 185 Fg^−1^ at this ratio, α-Fe_2_O_3_ had a capacitance of 699 Fg^−1^ by simple calculation, when a capacitance of 40 Fg^−1^ for pure PANI was considered. This simple calculation did not take into account the possibility of capacitance increase by PANI during the complexing process. Meanwhile, considering the ideal theoretical specific capacitance of α-Fe_2_O_3_ reported as 3625 Fg^−1^ [37,38], these results indicate that the capacitance of α-Fe_2_O_3_ can be maximized under the conditions in which the material surface is exposed as much as possible, and it is electrically connected effectively.

**Figure 6:**
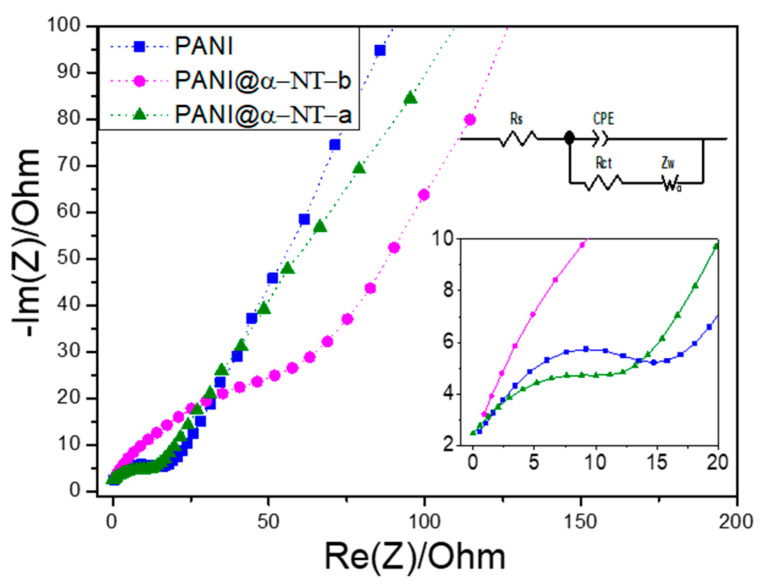
Nyquist electrochemical impedance spectra of PANI@α-NT-a, PANI@α-NT-b and PANI electrodes (inset: the enlarged high frequency region).

Quantitative EIS analysis was performed to investigate the resistive behavior in charge transfer due to the combination of PANI and α-NT (Figure 6). The electrode was prepared without the addition of carbon powder to exclude the effect of additional conducting materials. The electrolyte resistance (R_s_) and charge transfer resistance (R_ct_) at the interface between the electrode and electrolyte could be obtained from the Nyquist plots with the real impedance (Z). As shown in Figure 6, the R_ct_ obtained after fitting to an equivalent circuit model of “R_s_ + Q_ct_/(R_ct_ + Z_w_)” was 18 Ω for PANI, 14 Ω for PANI@α-NT-a, and 120 Ω for PANI@α-NT-b. Compared to PANI and PANI@α-NT-a, a relatively large R_ct_ appeared in PANI@α-NT-b. This can be attributed to the PANI content and the combined morphology of PANI and α-NT. The R_ct_ values of PANI and PANI@α-NT-a were almost the same, and it seems that the charge transfer through PANI was at the same level even in the complexed PANI@α-NT-a. Conversely, the 10 wt.% content of PANI in PANI@α-NT-b was insufficient to electrically connect the entire α-NT. These conditions influenced the surface redox inhibition, and the accumulation of ionic species was related to the decrease in capacitance. The result of showing a similar impedance spectrum in both PANI and PANI@α-NT-a, resulting in a large difference in capacitance, can be explained as a synergistic effect through the combination of PANI and α-NT. The electron transfer ability was improved by PANI, and the pseudocapacitance of α-Fe_2_O_3_ was utilized with high efficiency in PANI@α-NT-a, in which the inside and outside surfaces of α-NT were fully electrically connected.

**Figure 7:**
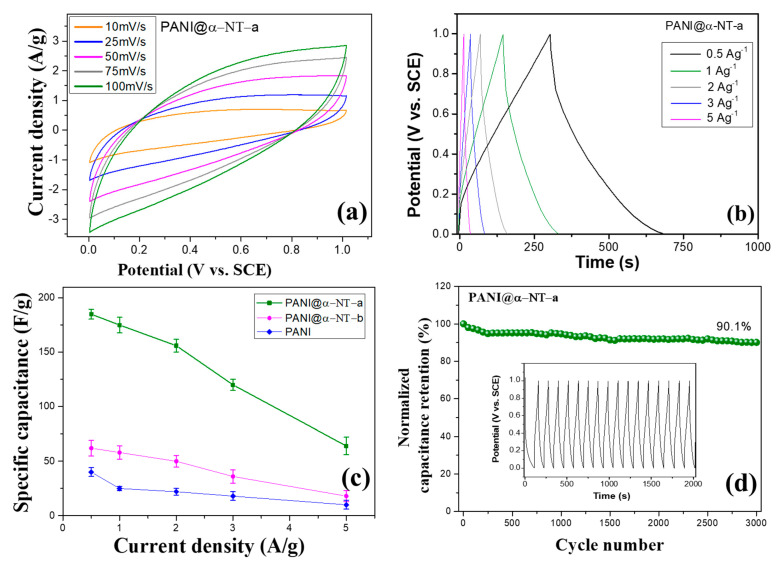
(**a**) Cyclic voltammetry (CV) curves for PANI@α-NT-a at different scan rates. (**b**) Charge–discharge curves for PANI@α-NT-a at different current densities. (**c**) The specific capacitance of PANI@α-NT-a, PANI@α-NT-b, and PANI electrodes at different current densities. (**d**) Cycling performance of PANI@α-NT-a electrode for 3000 cycles by GCD (the inset is GCD curves in the first 16 cycles).

The influence of the scan rate and applied current density on the capacitance of PANI@α-NT-a was investigated. The CV curves of PANI@α-NT-a showed that the current density gradually increased with an increasing scan rate between 10 and 100 mV s^−1^, maintaining the shape of the CV curve, indicating good reversibility at high scan rates (Figure 7a). The high current density during the charge/discharge process caused a capacitance decrease, which indicates that the low current response of the electrode material induces energy loss (Figure 7b). However, it seems that the reduction in capacitance is not only caused by the low-conductive α-NT part. The capacitance retentions at high current densities were 36%, 31%, and 30% (ratio of C_5.0 A/g_/C_0.5 A/g_) for PANI@α-NT-a, PANI@α-NT-b, and PANI, respectively, showing a similar decreasing level (Figure 7c). In addition, PANI@α-NT-a and PANI had similar R_ct_ in impedance analysis, and PANI@α-NT-b had a relatively large R_ct_, although all the cases showed very similar capacitance retention. From these results, it can be understood that the pseudocapacitance at high current density appears to be limited by not only the electrical conductivity of the materials, but also the charge accumulation in response to the applied current. It has been reported that the specific adsorption of sulfate anions (SO^2−^_4_) in electrolyte occurs at the surface sites of the oxide [39]. As a result, the limit of the charge balance interaction between the electrolyte and the electroactive surface, as well as the electrical conductivity of the electrode materials, determines the capacitance at a high current density.

The cyclic stability of PANI@α-NT-a was tested through GCD at 2.5 Ag^−1^ for 3000 cycles. Figure 7d shows the normalized capacitance retention with respect to cycle number. The specific capacitance continuously decreased until the initial 250 cycles, resulting in a cycle retention of 94.6%. After 250 cycles, it showed a very stable cycling stability and 90.1% retention after 3000 cycles. It is assumed that the initial capacitance reduction was due to the irreversible change and degradation affected by impurities or unstable chemical components of iron oxides and PANI. After this initial change, the capacitance was reduced very slowly, with only a 4.5% reduction up to 3000 cycles, confirming excellent electrode stability; PANI@α-NT-a provided superior overall cycling stability compared to the 79–87% capacitance retention reported for composites of PANI and metal oxides [40,41,42].

## 4. Conclusions

In summary, α-Fe_2_O_3_ nanotubes and their PANI-combined form of PANI@α-NTs were successfully prepared and tested for high-performance supercapacitor applications. The very low capacitance of α-NT was dramatically increased by the formation of combined structures with PANI. Thus, the specific capacitance of PANI@α-NT-b, in which PANI was coated inside the α-Fe_2_O_3_ nanotube, was 62 Fg^−1^, and PANI@α-NT-a containing PANI inside and outside α-Fe_2_O_3_ nanotubes showed a value of 185 Fg^−1^. PANI@α-NT-a exhibited a capacitance retention of 36% even when the current density was increased 10-fold, and showed excellent stability with 90.1% capacitance retention after 3000 charge–discharge cycles. In the EIS analysis, it was confirmed that the charge transfer resistance was minimized in PANI@α-NT-a, and the pseudocapacitance on the entire α-Fe_2_O_3_ nanotube surface was utilized with high efficiency through the binding and improvement of conductivity by PANI. These results will provide a route for the application of α-Fe_2_O_3_ as a supercapacitor electrode material by overcoming the inherent limitations of α-Fe_2_O_3_.

## Figures and Tables

**Table 1:** Specific capacitance of PANI@α-NTs, α-NTs and PANI at different current densities.

Electrode Materials	Specific Capacitance (Fg^−1^)					Weight % Ratio of the α-NT/PANI in the PANI@ α-NT
	Current Density (Ag^−1^)					
	0.5	1.0	2.0	3.0	5.0	
PANI@α-NT-a	185 (±4)	175 (±8)	156 (±7)	120 (±7)	66 (±8)	22/78
PANI@α-NT-b	62 (±8)	58 (±6)	50 (±5)	36 (±6)	19 (±5)	90/10
PANI	40 (±4)	25 (±2)	22 (±3)	18 (±5)	12 (±5)	0/100
α-NT	8.4 (±2)	-	-	-	-	100/0

## Data Availability

The data are included in the main text and the supplementary materials.

## Supplementary Materials

The following are available online at https://www.mdpi.com/article/10.3390/nano11061557/s1, Figure S1: Thermograms of thermogravimetric analysis for PANI@α-NTs.
