# Supplementary material for: Synergistic Effects of Fe2O3 Nanotube/Polyaniline Composites for an Electrochemical Supercapacitor with Enhanced Capacitance
Source: Nanomaterials (Basel). 2021 Jun 13;11(6):1557. doi: 10.3390/nano11061557 (PMC8231785; doi:10.3390/nano11061557)
Supplement: Supplementary file 1 [file nanomaterials-11-01557-s001.zip › nanomaterials-1230799-supplementary.pdf]

Supplementary Materials

Synergistic Effects of Fe_2O_3 Nanotube/Polyaniline Composites for an Electrochemical Supercapacitor with Enhanced Capacitance

Farkhod Azimov, Jihee Kim, Seong Min Choi and Hyun Min Jung

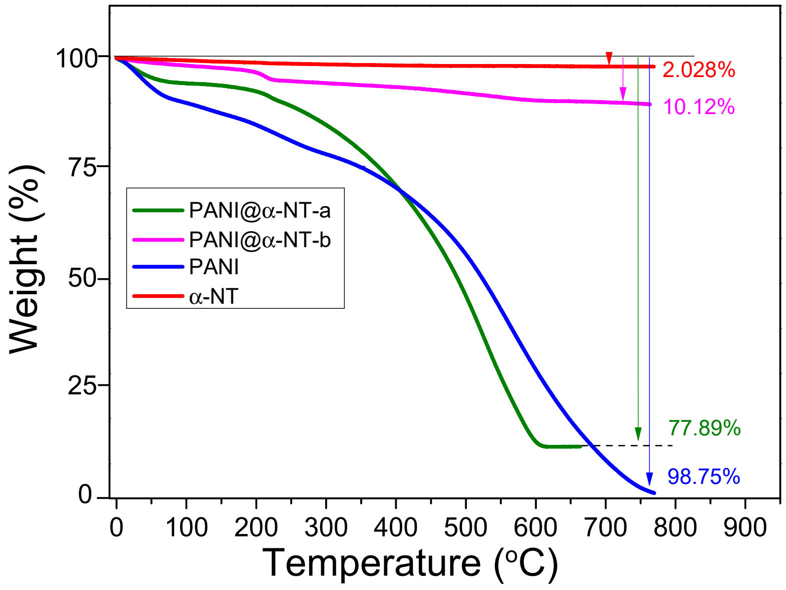

Figure S1. TGA curves of PANI@α-NT-a, PANI@α-NT-b, PANI and α-NT.
